# Impact of Simulation on Critical Care Fellows’ Electroencephalography Learning

**DOI:** 10.7759/cureus.24439

**Published:** 2022-04-24

**Authors:** Brenda G Fahy, Samsun Lampotang, Jean E Cibula, W. Travis Johnson, Lou Ann Cooper, David Lizdas, Nikolaus Gravenstein, Terrie Vasilopoulos

**Affiliations:** 1 Anesthesiology, University of Florida College of Medicine, Gainesville, USA; 2 Neurology/Epilepsy, University of Florida College of Medicine, Gainesville, USA; 3 Program Evaluation, University of Florida College of Medicine, Gainesville, USA; 4 Anesthesiology/Orthopedics and Rehabilitation, University of Florida College of Medicine, Gainesville, USA

**Keywords:** fellowship training, simulation, seizures, electroencephalography, critical care medicine

## Abstract

Introduction

Continuous electroencephalography (EEG) is an important monitoring modality in the intensive care unit and a key skill for critical care fellows (CCFs) to learn. Our objective was to evaluate with CCFs an EEG educational curriculum on a web-based simulator.

Methods

This prospective cohort study was conducted at a major academic medical center in Florida. After Institutional Review Board approval, 13 CCFs from anesthesiology, surgery, and pulmonary medicine consented to take an EEG curriculum. A 25-item EEG assessment was completed at baseline, after 10 EEG interpretations with a neurophysiologist, and after 10 clinically relevant EEG-based simulations providing clinical EEG interpretation hints. A 50-minute tutorial podcast was viewed after the baseline assessment. Main assessment outcomes included multiple outcomes related to web-based simulator performance: percent of hints used, percent of first words on EEG interpretation correct, and percent hint-based EEG interpretation score correct, with higher scores indicating more correct answers. Participants completed a 25-item EEG assessment before (baseline) and after the web-based simulator.

Results

All 13 CCFs completed the curriculum. Between scenarios, there were differences in percent of hints used (F_9,108_ = 11.7, p < 0.001), percent of first words correct (F_9,108 _= 13.6, p < 0.001), and overall percent hint-based score (F_9,108 _= 14.0, p < 0.001). Nonconvulsive status epilepticus had the lowest percent of hints used (15%) and the highest hint-based score (87%). Overall percent hint-based score (mean across all scenarios) was positively correlated with change in performance as the number of correct answers on the 25-item EEG assessment from before to after the web-based simulator activity (Spearman’s rho = 0.67, p = 0.023).

Conclusions

A self-paced EEG interpretation curriculum involving a flipped classroom and screen-based simulation each requiring less than an hour to complete significantly improved CCF scores on the EEG assessment compared to baseline.

## Introduction

In the intensive care unit (ICU), continuous electroencephalography (EEG) is an important monitoring modality. Accurate identification of EEG patterns that require intervention in a timely manner is a key skill for critical care fellows (CCFs) to acquire [1,2]. Continuous EEG monitoring in critically ill patients with primary neurological disorders, as well as non-neurological patients, revealed that over one-third of those monitored at some time showed seizure activity, with 10% presenting with nonconvulsive status epilepticus [3-5].

The Critical Care Continuous EEG Task Force of the American Clinical Neurophysiology Society consensus panel [6] noted that in critically ill adults and children, critical care continuous EEG monitoring (CCCEEG) plays an important role in detecting secondary injuries, including seizures and ischemia with altered mental status. The consensus panel “…recommends CCCEEG for diagnosis of nonconvulsive seizures, nonconvulsive status epilepticus, and other paroxysmal events, and assessment of the efficacy of therapy for seizures and status epilepticus. The consensus panel suggests CCCEEG for identification of ischemia in patients at high risk for cerebral ischemia; for assessment of the level of consciousness of patients receiving intravenous sedation or pharmacologically induced coma; and for prognostication in patients with cardiac arrest.” These recommendations [6] clearly encourage increased use of EEG as a monitoring and diagnostic tool in critically ill patients. Although EEG expertise is traditionally the domain of neurologists, CCFs from all backgrounds and especially non-neurology CCFs benefit from EEG exposure to gain a basic understanding and knowledge while providing care for their patients. In addition, familiarity with monitoring including EEG is an Accreditation Council for Graduate Medical Education requirement for CCFs [7]. Simulation can provide a reproducible learning platform when patient variability would be a confounding factor. Simulation is increasingly being used in education, including in brain death declaration [8]. Trainees can also have the opportunity to practice on simulators prior to instituting actual patient care, including just-in-time simulation training [9].

Using the expertise of a neurophysiology expert (JEC), we previously developed an interdisciplinary EEG curriculum using a flipped-classroom approach that entailed an educational podcast before class, followed by interpretation of EEGs with a neurologist during class, then a screen-based simulator approach for further learning that was completed outside the classroom. The curriculum was well-received by adult CCFs from varied training backgrounds, including anesthesiology, surgery, and internal medicine (pulmonary and critical care) [10]. This approach had comparable effectiveness to a previous cohort that had received traditional didactic and clinical training without the flipped classroom and screen-based simulation. This interactive simulator uses 10 clinically relevant cases with dynamic EEG tracings.

The purpose of this prospective cohort study was to further evaluate the impact of this EEG educational curriculum on performance on the screen-based simulator and assessment tools and to identify factors associated with better performance on the screen-based simulation and assessment tools. Secondary aims included evaluation of fellows’ podcast and technology experiences to better understand how previous experiences with podcasts and technology may impact learning that uses simulation as a teaching strategy.

## Materials and methods

**Figure 1:**
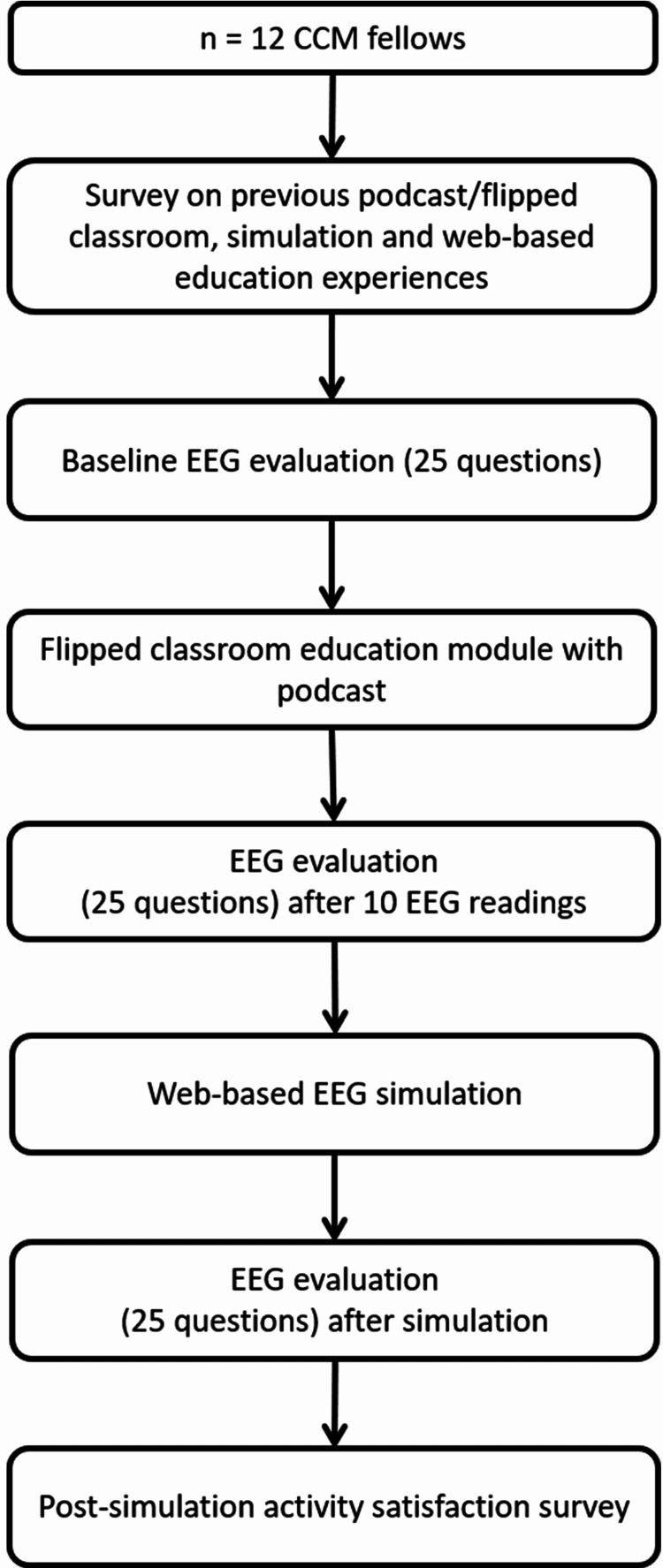
Flow chart for the study protocol. CCM, critical care medicine; EEG, electroencephalography.

The University of Florida Institutional Review Board (IRB201701046) approved this study and 13 of the CCFs who rotated through a neurocritical care unit consented to participate in the EEG educational curriculum. After enrollment, the CCFs took a survey on their previous podcast/flipped classroom, simulation, and web-based educational experiences and technology assessment tool, as previously described [11]. They also took a baseline 25-item multiple-choice EEG interpretation assessment, which included EEG interpretations (Figure 1).

The actual curriculum began with participants watching a 50-minute EEG podcast, which covered the basics of EEG as described previously [12]. It included technological and monitoring aspects of EEG, normal awake and sleep patterns, and EEG patterns that are important to recognize clinically (e.g., status epilepticus). After the podcast, the participants interpreted 10 EEGs under the guidance of a neurophysiologist. This was followed by the completion of 10 additional clinically relevant ICU scenarios on a previously described [13] web-based EEG simulator using clinical vignettes and corresponding dynamic EEG tracings.

The simulation EEG tracing automatically pauses at appropriate points and a question is displayed for up to 1 minute, requiring an answer entry in a free-text box. When answered correctly, the simulation scenario proceeded to the next question point or a new clinical vignette began. When answered incorrectly, one or more hints were provided, with an additional 30 seconds allowed for the learner to enter an answer after a hint. Then the simulation continued with the correct answer entry. Up to two hints were available for each question after an incorrect answer entry. If the available hints were exhausted, the answer was displayed and participants needed to type that correct answer in the free-text box to continue the simulation. The simulator design allowed the participant to suspend the session and resume where they had stopped. The main assessment outcomes for each scenario included percent of hints used (number of hints used/number of hints available), percent of correct first words (number of correct answers on the first try/total questions), and percent hint-based score, with correct answers weighted by the number of hints used. Higher scores indicated more correct answers using fewer hints (total hint-based score obtained/maximum hint-based score). After the 10 EEG simulation scenarios, the CCFs completed another 25-item EEG assessment. Additionally, CCFs completed a survey on the previous podcast/flipped classroom, simulation, and web-based educational experiences that have been described previously [11].

Simulation performance metrics were summarized as mean (standard deviation), along with 95% CIs for each scenario and overall. Repeated measures ANOVA was used to assess between scenario differences in performance; a repeated measures approach was used to account for the same fellows completing each scenario. To assess the reliability of scenarios, Cronbach α was calculated by using an average percent hint-based score from each scenario. One-way ANOVA was also used to evaluate percent hint-based score differences across CCF training programs (anesthesiology, pulmonary, and surgery). Welch’s correction was performed for unequal variances. The overall change in the 25-item EEG assessment as the number of correct answers before (baseline) and after web-based simulator activity was evaluated using a paired t-test, with a statistically significant result supporting improvement following the simulator activity. Spearman’s correlations (rho) were calculated to assess the association between improvement in the 25-item EEG assessment and percent hint-based scores in the web-based simulator activity. They were calculated for overall hint-based score (mean across all scenarios) and separately for hint-based scores of each scenario, with bootstrapped 95% CIs. For correlation analyses, change in the 25-item EEG assessment was calculated as a residual change score from before (baseline) and after web-based EEG simulator activity, which accounts for participant differences at baseline. p < 0.05 was considered statistically significant. JMP Pro 15 (SAS Institute Inc, Cary, NC) was used for analysis.

## Results

Thirteen (n = 13) CCFs completed the web-based EEG simulator; six fellows were from an anesthesiology background, four from surgery, and three from pulmonary medicine. Twelve completed the previous podcast/flipped classroom experiences survey. The CCF baseline performance on the 25-item EEG assessment was 10.4 ± 3.0 correct interpretations.

**Figure 2:**
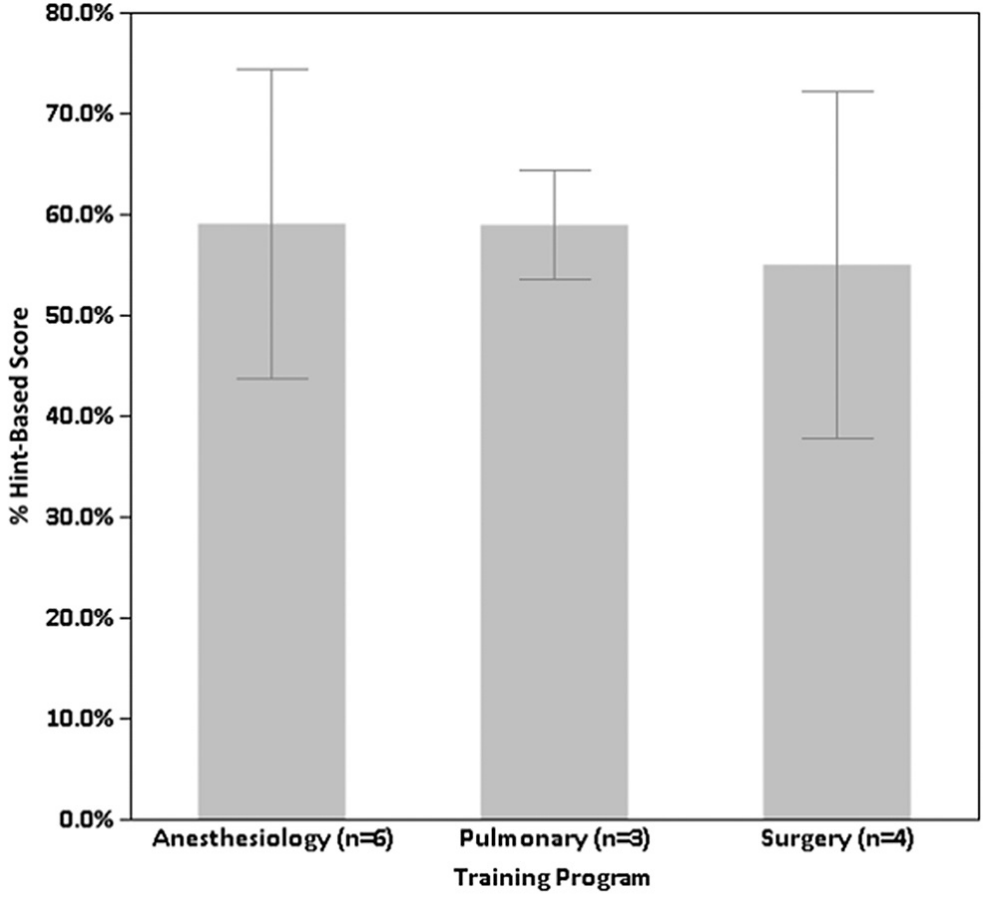
Association between critical care training program and the overall percent of the hint-based score. Error bars indicate 95% CIs.

Table 1 reports the percent of hints used, percent of the first-word score, and percent of the hint-based score for each scenario, as well as overall. Scenarios demonstrated overall acceptable reliability (Cronbach α = 0.75). There were statistically significant between scenario differences in percent of hints used (F_9,108_ = 11.7, p < 0.001), percent of first words correct (F_9,108_ = 13.6, p < 0.001), and overall percent hint-based score (F_9,108_ = 14.0, p < 0.001, Table 1). For percent of hints used, scenario 10 (nonconvulsive status epilepticus) had the lowest percent of hints used (15%), whereas scenarios three (burst suppression and medication effect), four (focal epileptogenic potentials), and eight (primary generalized epilepsy) had more than 60% of hints used. Scenario 10 (nonconvulsive status epilepticus) also had the highest percent first-word score (86%) and scenario four (focal epileptogenic potentials and slowing) had the lowest (17%). For percent hint-based score, scenario 10 (nonconvulsive status epilepticus) again had the highest score (87%), whereas scenarios one (normal EEG and artifact), three (burst suppression and medication effect), four (focal epileptogenic potentials and slowing), and eight (primary generalized epilepsy) had scores of less than 60%. There were no statistically significant differences in the overall percent of hint-based scores across training programs (F_2,10_ = 0.16, p = 0.86, Figure 2).

**Table 1:** Electroencephalography Performance by Scenario EEG, electroencephalography.

Scenario	N	Total Hints Available	Hints Used (%) Mean (SD) 95% CI	Number of Questions	First Word Correct (%) Mean (SD) 95% CI	Maximum Overall Score	Overall Score (%) Mean (SD) 95% CI
1. Normal EEG and artifact	13	20	48.8 (22.9) 35.0, 62.7	11	46.2 (19.8) 34.2, 58.1	88	54.2 (23.7) 39.8, 68.5
2. Focal onset seizures	13	8	35.6 (27.9) 18.7, 52.4	4	59.6 (29.8) 41.6, 77.6	32	64.4 (27.9) 47.5, 81.3
3. Bust suppression and medication effect	13	10	64.6 (17.6) 54.0, 75.3	6	29.5 (12.1) 22.9, 36.8	48	36.2 (16.5) 26.2, 46.2
4. Focal epileptogenic potentials and showing	13	6	68.0 (25.9) 52.3, 83.6	4	17.4 (23.7) 3.0, 31.6	32	32.7 (25.3) 17.4, 48.0
5. Coma and postanoxic myoclonus including artifacts	13	8	42.3 (21.4) 29.4, 55.2	5	53.8 (22.2) 40.4, 67.3	40	63.1 (18.9) 51.7, 74.5
6. Cerebral ischemia	13	19	32.8 (14.9) 23.8, 41.8	10	60.8 (13.8) 52.4, 69.1	80	68.7 (15.5) 59.3, 78.0
7. Sleep and sedative effects	13	13	37.9 (15.9) 28.3, 47.4	8	49.0 (11.9) 41.8, 56.2	64	69.5 (12.8) 61.8, 77.2
8. Primary generalized epilepsy	13	11	65.7 (13.5) 57.6, 73.9	8	29.5 (19.4) 17.7, 41.2	48	34.0 (12.5) 26.4, 41.5
9. Brain death/isoelectric EEG	13	6	32.1 (27.6) 15.4, 48.7	3	51.3 (25.9) 35.6, 66.9	24	67.9 (27.6) 51.3, 84.6
10. Nonconvulsive status epilepticus	13	18	15.4 (9.7) 9.5, 21.2	14	85.7 (8.2) 80.1, 90.7	112	87.1 (8.2) 82.1, 92.0
Scenario overall	13		44.3 (11.3) 37.5, 51.1		48.3 (8.3) 43.2, 53.3		57.8 (11.1) 51.1, 64.5

Nine fellows reported at least four previous prior podcast educational experiences and six reported 10 or more previous experiences with educational podcasts. Only 25% of fellows reported fewer than four previous experiences with any type of web-based education. For simulation specifically, eight fellows reported four or more previous educational experiences involving simulations. Those with less previous simulation education experience (<4 times; mean [n = 4] = 59.1%, 95% CI: 46.2%, 72.1%) did not have a significantly different overall percent hint-based scores compared to those with more previous experience (≥ 4 times; mean [n = 8] = 55.8%, 95% CI: 46.7%, 65.0%) [t(9.7) = 0.60, p = 0.56].

**Figure 3:**
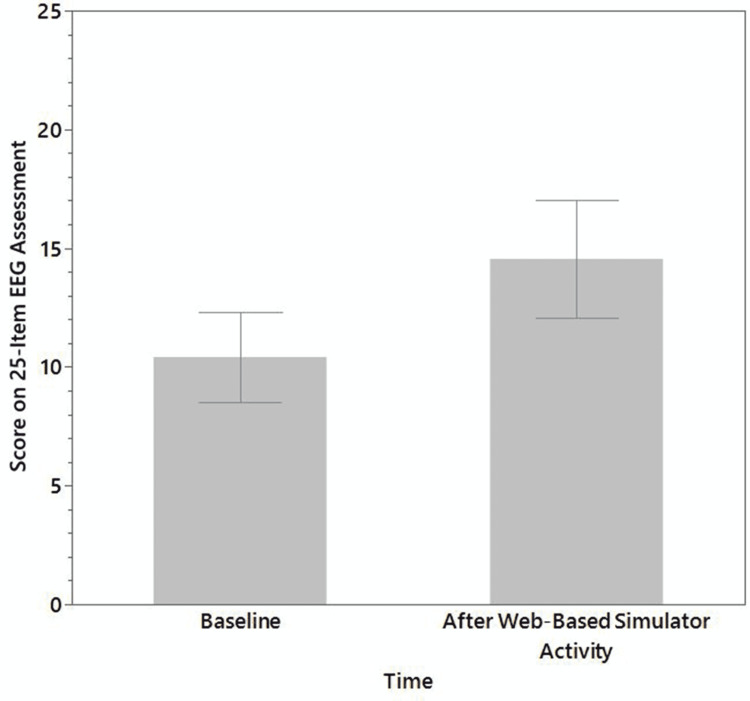
Overall improvement in 25-item EEG assessment as represented by the number of correct answers from before (baseline) to after web-based simulator activity. Error bars indicate 95% CIs.

Figure 3 presents overall performance on the 25-item EEG assessment before and after web-based simulator activity. Participant scores on the 25-item EEG assessment significantly improved (p = 0.012) following the web-based simulator activity (mean change = 3.9, 95% CI: 1.1, 6.7). Table 2 shows correlations between residual change scores in performance on the 25-item EEG assessment and percent hint-based scores on the web-based simulator activity. Improvement in scores on the 25-item EEG assessment before and after the web-based simulator activity was positively correlated with the overall percent hint-based score (rho = 0.67, p = 0.023), indicating that higher overall hint-based scores on the simulation were associated with improvement in the EEG assessment. For separate scenarios, improvement in scores on the 25-item EEG assessment were positively correlated with higher hint-based scores on scenarios involving normal EEG artifact (rho = 0.69, p = 0.018), focal onset seizures (rho = 0.71, p = 0.014), focal epileptogenic potentials and showing (rho = 0.70, p = 0.016), and primary generalized epilepsy (rho = 0.77, p = 0.006).

**Table 2:** Spearman’s correlations between percent of hint-based scores and change in performance on 25-item EEG assessment from baseline to after simulation. EEG, electroencephalography.

Scenario	Spearman’s Correlation	Bootstrapped 95% CI	P-value
1. Normal EEG and artifact	0.69	0.10, 0.98	0.018
2. Focal onset seizures	0.71	0.05, 0.93	0.014
3. Bust suppression and medication effect	–0.03	–0.72, 0.62	0.936
4. Focal epileptogenic potentials and showing	0.70	0.05, 0.98	0.016
5. Coma and postanoxic myoclonus including artifacts	–0.25	–0.94, 0.54	0.463
6. Cerebral ischemia	–0.19	–0.79, 0.60	0.581
7. Sleep and sedative effects	0.59	0.09, 0.91	0.057
8. Primary generalized epilepsy	0.77	0.37, 0.95	0.006
9. Brain death/isoelectric EEG	0.38	–0.29, 0.95	0.246
10. Nonconvulsive status epilepticus	0.22	–0.63, 0.86	0.523
Scenario overall	0.67	0.10, 0.99	0.023

## Discussion

EEG is an increasingly important diagnostic and prognostic monitor in the ICU. One example of the effect of CCCEEG monitoring is early detection of nonconvulsive status epilepticus, which enables rapid treatment of this neurological emergency. Often, treatment for this emergent condition is delayed because of its nonconvulsive presentation [14,15]. EEG monitoring is also useful with altered mental status, cerebral ischemia, differentiating post-anoxic myoclonus from seizure, achievement of burst suppression, and as a confirmatory test for brain death.

Our study demonstrated that a brief interdisciplinary EEG curriculum involving a flipped classroom and screen-based simulation significantly improved EEG knowledge based on performance on the EEG assessment among CCFs from various backgrounds. Improvement on our EEG assessment was correlated not only with overall simulation performance but also with performance in several specific scenarios, mainly ones involving normal EEG and EEGs related to seizures and epilepsy. This provides support that our web-based simulations, especially with these specific scenarios, supported learning. However, not all scenarios were associated with improvement in the EEG assessment; furthermore, there was variability in overall performance across scenarios. This provides valuable information on specific areas of need for enhancement of the EEG training curricula. The educational approach of this hybrid EEG curriculum can be adapted to the particular needs of trainees from varying backgrounds. This curriculum was designed to provide basic and clinically relevant knowledge for managing critically ill patients monitored by CCCEEG and was not intended to be comprehensive training for EEG interpretation.

Important considerations for this curriculum are how will it translate to patient care and whether there is long-term retention. We have previously reported on trainee satisfaction and impressions of our simulation curriculum [11]. A majority of CCFs (90%) reported that the simulator improved both their confidence and perceived ability in performing EEG interpretations and in making treatment decisions. This finding, combined with the present study’s finding that better simulator performance was associated with better EEG knowledge, provides support that this curriculum will positively contribute to patient care involving EEGs. Although we did not examine long-term retention in the present study, our research group’s previous work with other flipped-classroom models and EEG knowledge has shown that improvements in EEG knowledge were retained 12 months following the flipped-classroom EEG curriculum [16].

The training in this study was required as part of the education for CCFs for neurocritical care. Current medical trainees are adult learners who are familiar with electronic communications and prefer independent learning options [17,18]. A graduate medical education program in general surgery compared a traditional in-person curriculum to an online curriculum attendance and found that the online curriculum significantly increased participation [19]. In a systemic review of flipped classrooms in graduate medical education, trainees expressed a positive attitude toward flipped-classroom learning [20]. Additionally, we previously found that neurology residents in our training program predominantly had a kinesthetic learning preference, which is consistent with an interactive learning preference [21]. From one of our earlier studies with residents and medical students [11], nearly half of the participants reported having fewer than four previous educational experiences with podcasts. In the current study, 75% of participants reported at least four previous podcast experiences, and 50% reported at least 10 previous experiences. Furthermore, nearly all of the participants in the current study reported at least four previous experiences with some type of web-based education.

This EEG curriculum attempted to use higher levels of learning by applying principles learned to a simulated clinical situation. This curriculum used self-directed learning, as well as spaced learning between curriculum activities, which contribute to long-term retention [22]. The interactive EEG simulations were designed to use the adult learning principle of active retrieval. This principle involves the learner having to do additional processing and retrieve information repeated with the handling of information; this approach is more likely to lead to learning and retention of the knowledge [23,24]. The simulation was designed to require free-text entry; thus, it required more retrieval practice than multiple choice where an answer is selected from a list of options and guessing maybe factor [25].

Although learners had different primary backgrounds, limitations include that all were from a single institution university-based program, constituting a small sample size. Thus, the sample may not be representative of all CCFs and it was underpowered to detect smaller differences in performance on simulation. Other measurements of performance including technical and non-technical skills were not evaluated, recognizing that associations may be different from other performance measurements.

## Conclusions

This pilot study provides evidence that an interdisciplinary EEG curriculum with simulation can be an effective educational method as evaluated by an EEG interpretation assessment tool to impart EEG knowledge to CCFs from different backgrounds (anesthesiology, surgery, and pulmonary medicine). EEG abnormalities with lower EEG interpretation scores indicate opportunities for future education and study. Further studies are required to explore the impact of better EEG interpretation skills on patient outcomes.
